# Impact of “3414” fertilization on the yield and quality of greenhouse tomatoes

**DOI:** 10.1515/biol-2022-0893

**Published:** 2024-06-28

**Authors:** Chunyan Wu, Xiaoyi Han, Yan Cheng, Xueke Wang, Wei Wang

**Affiliations:** College of Horticulture, Jilin Agricultural University, Changchun, Jilin, 130118, China; Economic Botany Institute, Jilin Academy of Agricultural Sciences, Changchun, Jilin 130033, China

**Keywords:** “3414” experimental design, quality, tomato, yield

## Abstract

This study aimed to explore the effects of different nitrogen, phosphorus, and potassium ratios on the yield and nutritional quality of greenhouse tomatoes under a water and fertilizer integration model. Greenhouse tomatoes were used as the research object, and the “3414” fertilizer trial design was employed to assess tomato growth, yield, quality, and soil indicators across various treatment combinations. The goal was to determine the optimal fertilization scheme and recommend appropriate fertilizer quantities for tomato cultivation and production. The results revealed that different fertilizer ratios significantly affected both the quality and yield of tomatoes. Overall, the tomato yield tended to increase with higher fertilization amounts, with potassium exhibiting the most pronounced effect on yield increase, followed by phosphorus and nitrogen. The comprehensive analysis of principal components indicated that the N_2_P_2_K_1_ treatment yielded the highest nutritional quality and yield. Therefore, the best fertilization combination identified in this study consisted of nitrogen fertilizer at 197.28 kg hm^−2^, phosphorus fertilizer at 88.75 kg hm^−2^, and potassium fertilizer at 229.80 kg hm^−2^. These findings provided the scientific basis for optimizing fertilization practices in greenhouse tomato cultivation and production in the Jilin Province.

## 1 Introduction

Tomato (*Lycopersicon esculentum*) is an annual or perennial herbaceous plant belonging to the Solanaceae family. It is extensively cultivated in both northern and southern regions of China. It ranks among the most commonly grown greenhouse vegetables globally [1]. Tomatoes are characterized by their vibrant colors, distinctive flavor, and high contents of nutrients, such as carotene, lycopene, and vitamins C and B. Besides their nutritional value, tomatoes possess high medicinal value, aiding digestion, detoxification, and preventing cardiovascular diseases [2,3]. Given their high water and nutrient requirements, greenhouse tomato cultivation has gained widespread adoption, ensuring a steady supply of fresh produce. Integrated water and fertilizer technologies represent advanced fertilizer management techniques that enhance water and nutrient utilization efficiency, ultimately maximizing economic returns [4,5]. Fertilization is a pivotal technique in tomato cultivation and production, playing a crucial role in improving soil fertility, facilitating tomato growth and development, and increasing yield and quality [6,7]. However, the indiscriminate use of fertilizers can have negative consequences [8,9]. Therefore, appropriate fertilization practices are essential for promoting agricultural productivity and achieving superior crop quality and yield.

Nitrogen, phosphorus, and potassium are essential elements for the normal growth and development of plants. Improper combinations of these three elements can lead to crop nutrient imbalances, increased fruit nitrate content, decreased quality, low yield, and imbalanced soil nutrient supply [10,11,12]. Studies on the impact of nitrogen, phosphorus, and potassium fertilizers on tomatoes exist [13,14]. However, previous studies have examined only a limited range of nitrogen, phosphorus, and potassium ratios, neglecting the interactive effects and influence of multiple levels. In addition, the variations in soil fertility, crop residues, and cultivated varieties can alter the optimal fertilization scheme for crops. The “3414” experimental design plays a significant role in national soil testing and formula fertilization efforts. It can determine the appropriate application amounts of nitrogen, phosphorus, and potassium based on soil fertility, crop fertilization requirements, and fertilizer response functions. This experimental plan is extensively used for studying fertilizer efficiency both domestically and internationally [15]. The high-quality and efficient cultivation of greenhouse tomatoes in the Jilin Province holds significant economic importance for greenhouse film industries. This study was based on the integrated water and fertilizer technology for tomatoes and aimed to strengthen the research on rational fertilization techniques for greenhouse tomatoes. Employing the “3414” experimental design, we investigated the combined effects of nitrogen, phosphorus, and potassium application on the growth and quality of tomatoes in greenhouse facilities. The goal was to determine the optimal fertilization ratio to improve the tomato yield and quality, providing a theoretical basis for scientifically sound fertilization practices in high-quality tomato production and cultivation.

## 2 Materials and methods

### 2.1 Study location and materials

The study was conducted at the teaching and research base of Jilin Agricultural University, situated at 125°41′ East longitude and 43°82′ North latitude. The area experiences a temperate continental semi-humid monsoon climate characterized by an average annual sunshine duration of 2544.8 h, an effective accumulated temperature of 2,850℃, a frost-free period of 145 days, an average annual temperature of 5.8℃, and an average annual precipitation of 558 mm. The soil used in the study was black soil, possessing the following basic physical and chemical properties: bulk density of 0.97 g cm^−3^, total porosity of 59.66%, soil pH of 6.7, organic matter content of 34.39 g kg^−1^, alkali nitrogen content of 161 mg kg^−1^, available phosphorus content of 50.91 mg kg^−1^, and available potassium content of 190.04 mg kg^−1^. The tomato variety selected for the experiment was “Shidun 197,” characterized by an indeterminate growth type with medium early maturity. The fertilizers used included urea (N: 46%), calcium superphosphate (P_2_O_5_: 12%), and potassium sulfate (K_2_O: 50%).

### 2.2 Study design

The tomato seeds were sown in March 2023, using pot planting experiments, and later transplanted on May 6, with 5 plants per treatment and 3 repetitions, totaling 210 plants. During the growth period, single-stem pruning was conducted, with five fruit clusters retained per plant. The harvesting started on July 15, 2023. The study employed the “3414” fertilizer trial plan, with nitrogen, phosphorus, and potassium serving as the 3 elements, each at 0, 1, 2, and 3 levels, resulting in 14 treatments. The experimental factors and levels are detailed in Table 1, and the experimental treatments and corresponding fertilization amounts are provided in Table 2. Fertilization started 10 days after planting, facilitated by a fertigation system for integrated irrigation and fertilization management. The fertilizer application adhered to the critical growth periods of tomatoes, with 15% of the total supply administered during the planting-to-flowering phase and 85% during the fruiting period. Additionally, the water supply was adjusted following tomato growth and prevailing weather conditions.

**Table 1:** Nitrogen, phosphorus, and potassium fertilization levels

Fertilizer level code	N (kg hm^−2^)	P_2_O_5_ (kg hm^−2^)	K_2_O (kg hm^−2^)
0	0.00	0.00	0.00
1	98.64	44.38	229.80
2	197.28	88.75	459.60
3	295.92	133.13	689.40

**Table 2:** Experimental treatments and fertilizer application amount

Treatment	Fertilizer application amount (kg hm^−2^)		
	N	P_2_O_5_	K_2_O
N0P0K0	0.00	0.00	0.00
N0P2K2	0.00	88.75	459.60
N1P2K2	98.64	88.75	459.60
N2P0K2	197.28	0.00	459.60
N2P1K2	197.28	44.38	459.60
N2P2K2	197.28	88.75	459.60
N2P3K2	197.28	133.13	459.60
N2P2K0	197.28	88.75	0.00
N2P2K1	197.28	88.75	229.80
N2P2K3	197.28	88.75	689.40
N3P2K2	295.92	88.75	459.60
N1P1K2	98.64	44.38	459.60
N1P2K1	98.64	88.75	229.80
N2P1K1	197.28	44.38	229.80

Note: The N_0_P_0_K_0_ treatment in the table serves as the control treatment (CK).

### 2.3 Measurement indicators and methods

The soil pH value was measured using the potentiometric method with a pHS-3E acidity meter (Shanghai Yoke Instrument Co., Ltd.). The electrical conductivity (EC) value was determined using the conductivity method with a DDS-11A electromagnetic conductivity meter (Shanghai Yoke Instrument Co., Ltd.). The alkali nitrogen content was determined using the NaOH alkali diffusion–absorption method. The available phosphorus content was determined using the NaHCO_3_ extraction–molybdenum antimony colorimetric method. The available potassium content was determined using the NH_4_OAC extraction-flame photometry method [16].

The plant height was measured using a tape measure from the first leaf to the top growth point. The stem thickness was measured using a Vernier caliper at the midpoint between the first and second leaves. The tomato leaf area was obtained using the weighing method. Starting from the tomato harvest period, the yield for each experimental treatment was determined, and the yield per plant was recorded.

The soluble sugar content of tomato fruit was measured using the anthrone method. The soluble protein content was determined using the Coomassie brilliant blue G-250 staining method. The soluble solid contents were measured using a refractometer. The organic acid contents were determined using acid–base titration. The vitamin C content was determined using the molybdenum blue colorimetric method. The nitrate and lycopene contents were determined using ultraviolet spectrophotometry [17,18].

### 2.4 Statistical analysis

The experimental data were organized using Microsoft Excel 2021. Significance testing and principal component analysis were conducted using SPSS version 26 statistical analysis software.

## 3 Results

### 3.1 Effects of different nitrogen, phosphorus, and potassium ratios on the physical and chemical properties of soil for tomato cultivation

The physical and chemical properties of the experimental soil under different fertilization treatments are presented in Table 3. The soil pH under the CK treatment was the highest (7.39), significantly surpassing that under other treatments. Conversely, the pH of the N_3_P_2_K_2_ treatment was the lowest (6.32), indicating that the high-nitrogen fertilizer treatment resulted in decreased pH levels. This decline in soil pH could be attributed to long-term excessive fertilization. The EC value was the highest under the N_3_P_2_K_2_ treatment (1145.33 µs cm^−1^), significantly exceeding that under other treatments. Conversely, the N_0_P_2_K_2_ treatment exhibited the lowest EC value (317.23 µs cm^−1^), indicating that the application of higher amounts of fertilizer led to increased soil EC values. This elevation implied a higher salt content in the soil, potentially impeding plant growth and development. The highest alkali nitrogen content was recorded under the N_3_P_2_K_2_ treatment (172.67 mg kg^−1^), followed by that under the N_2_P_2_K_3_ and N_2_P_2_K_2_ treatments (169.17 and 166.83 mg kg^−1^), respectively.

**Table 3:** Effects of different nitrogen, phosphorus, and potassium ratios on the physicochemical properties of soil for tomato cultivation

Treatment	pH	EC (µs cm^−1^)	Alkaline nitrogen (mg kg^−1^)	Available phosphorus (mg kg^−1^)	Available potassium (mg kg^−1^)
N0P0K0	7.39 ± 0.01a	436.00 ± 3.06f	114.33 ± 1.17g	35.82 ± 0.25f	160.11 ± 0.86fg
N0P2K2	7.20 ± 0.01b	317.23 ± 0.84h	121.33 ± 3.09f	40.36 ± 0.67def	208.85 ± 1.71c
N1P2K2	7.01 ± 0.01d	579.33 ± 41.70d	128.33 ± 2.33ef	44.34 ± 0.67cd	211.42 ± 0.86c
N2P0K2	6.93 ± 0.01e	342.80 ± 1.65h	135.33 ± 1.17de	38.54 ± 1.75ef	219.11 ± 2.26b
N2P1K2	7.10 ± 0.01c	732.00 ± 4.16c	151.67 ± 1.17b	39.80 ± 1.56def	195.17 ± 1.48d
N2P2K2	7.00 ± 0.01d	458.33 ± 8.88f	166.83 ± 4.67a	46.51 ± 0.71bc	183.20 ± 1.71e
N2P3K2	6.72 ± 0.03f	813.33 ± 6.74b	156.33 ± 2.33b	54.13 ± 1.68a	182.35 ± 2.96e
N2P2K0	7.12 ± 0.02c	451.67 ± 8.65f	138.83 ± 1.17cd	41.06 ± 1.65de	158.40 ± 0.86g
N2P2K1	6.66 ± 0.02f	378.17 ± 7.19g	138.83 ± 3.07cd	44.48 ± 0.92 cd	164.39 ± 2.57fg
N2P2K3	6.65 ± 0.01f	589.67 ± 2.03d	169.17 ± 1.17a	52.31 ± 1.78a	230.23 ± 0.86a
N3P2K2	6.32 ± 0.03g	1145.33 ± 3.93a	172.67 ± 1.17a	50.00 ± 2.58ab	190.90 ± 1.71d
N1P1K2	7.10 ± 0.01c	523.67 ± 2.19e	128.33 ± 1.17ef	49.38 ± 1.21ab	222.53 ± 3.08b
N1P2K1	6.92 ± 0.03e	452.33 ± 2.33f	128.33 ± 3.07ef	51.05 ± 2.47ab	165.24 ± 3.08f
N2P1K1	6.88 ± 0.06e	398.03 ± 4.01g	143.50 ± 2.02c	39.10 ± 0.53ef	195.17 ± 2.57d

Note: The data in the table represent mean ± standard deviation. Different letters following the numbers in the same column indicate significant differences at the 0.05 level.

The lowest alkali nitrogen content was recorded under the CK treatment (114.33 mg kg^−1^), indicating that applying nitrogen fertilizers increases the alkali nitrogen content in the soil. The available phosphorus content was the highest under the N_2_P_2_K_3_ treatment (54.13 mg kg^−1^), followed by that under the N_2_P_2_K_3_ treatment (52.31 mg kg^−1^), with no significant difference compared with that under N_1_P_2_K_1_, N_3_P_2_K_2_, and N_1_P_1_K_2_ treatments but with significant difference compared with that under other treatments. Conversely, the available phosphorus content was the highest under the CK treatment (35.82 mg kg^−1^), indicating that soil without phosphorus fertilizers had a lower available phosphorus content. Moreover, the available potassium content was the highest under the N_2_P_2_K_3_ treatment (230.23 mg kg^−1^), which was significantly higher than that under other treatments. Conversely, the available potassium content was the lowest under the N_2_P_2_K_0_ treatment (158.40 mg kg^−1^), indicating that applying potassium fertilizers significantly increases the available potassium content in the soil.

### 3.2 Effects of different nitrogen, phosphorus, and potassium ratios on the growth indicators of tomato plants

As depicted in Table 4, the tallest plant was observed under the N_0_P_2_K_2_ treatment (133.23 cm), followed by 132.10 cm under the CK treatment. Conversely, the plant heights under N_2_P_2_K_2_, N_1_P_2_K_1_, N_1_P_2_K_2_, and N_1_P_1_K_2_ treatments were significantly lower than those under N_0_P_2_K_2_ and CK treatments. The plants were the shortest under the N_1_P_1_K_2_ treatment (94.30 cm). The CK treatment, with no fertilizer, led to better plant growth, indicating that the soil fertilizers used during the experiment ensured the normal growth of tomatoes in the early stage. Additionally, using equivalent nitrogen fertilizer amounts, the combined application of phosphorus and potassium fertilizers positively impacted the plant growth.

**Table 4:** Effects of different nitrogen, phosphorus, and potassium ratios on tomato growth indexes

Treatment	Plant height (cm)	Stem diameter (mm)	Leaf area (cm^2^)	Dry matter accumulation (g/plant)
N_0_P_0_K_0_	132.10 ± 0.25a	11.51 ± 0.28abc	72.07 ± 1.65abcde	194.47 ± 2.02d
N_0_P_2_K_2_	133.23 ± 5.75a	11.96 ± 0.94ab	74.44 ± 1.40abcd	240.10 ± 0.63c
N_1_P_2_K_2_	95.13 ± 10.49b	10.06 ± 1.01bcd	63.53 ± 1.80e	244.83 ± 7.68c
N_2_P_0_K_2_	117.60 ± 3.40ab	11.15 ± 0.64abcd	79.73 ± 2.27a	237.51 ± 12.13c
N_2_P_1_K_2_	118.93 ± 12.79ab	11.38 ± 0.38abc	65.51 ± 4.84de	247.90 ± 7.32c
N_2_P_2_K_2_	95.97 ± 6.87b	9.34 ± 0.31d	70.22 ± 2.49abcde	281.12 ± 2.37b
N_2_P_3_K_2_	109.57 ± 6.18ab	9.24 ± 0.20d	71.24 ± 2.33abcde	198.83 ± 5.26d
N_2_P_2_K_0_	114.57 ± 11.31ab	10.76 ± 0.43abcd	64.92 ± 1.42de	208.10 ± 2.17d
N_2_P_2_K_1_	106.13 ± 12.37ab	11.79 ± 0.87ab	70.99 ± 6.48abcde	298.89 ± 3.12a
N_2_P_2_K_3_	101.87 ± 10.69ab	10.15 ± 0.23bcd	77.04 ± 3.06ab	232.50 ± 3.07c
N_3_P_2_K_2_	121.07 ± 9.76ab	11.30 ± 0.65abc	67.52 ± 1.81bcde	199.03 ± 6.10d
N_1_P_1_K_2_	94.30 ± 8.99b	10.87 ± 0.35abcd	67.16 ± 2.32cde	206.41 ± 5.88d
N_1_P_2_K_1_	95.60 ± 4.57b	9.63 ± 0.74 cd	66.12 ± 1.48de	195.62 ± 3.30d
N_2_P_1_K_1_	112.40 ± 15.20ab	12.13 ± 0.10a	76.59 ± 1.63abc	310.29 ± 5.94a

Note: The data in the table represent mean ± standard deviation. Different letters following the numbers in the same column indicate significant differences at the 0.05 level.

The stem thickness was the highest under the N_2_P_1_K_1_ treatment (12.13 mm), significantly differing from that under N_2_P_2_K_3_, N_1_P_2_K_2_, N_1_P_2_K_1_, N_2_P_2_K_2_, and N_2_P_3_K_2_ treatments. This represented a 5.39% increase compared with the CK treatment. Conversely, the stem thickness was the lowest under the N_2_P_3_K_2_ treatment (9.24 mm), significantly differing from that under N_3_P_2_K_2_, N_2_P_1_K_2_, CK, N_2_P_2_K_1_, N_0_P_2_K_2_, and N_2_P_1_K_1_ treatments. These findings suggested that the application of appropriate phosphorus and potassium amounts could enhance tomato stem thickening within a certain range.

The leaf area was the largest under the N_2_P_1_K_1_ treatment (79.72 cm²), followed by 77.04 and 76.59 cm² under the N_2_P_2_K_3_ and N_2_P_1_K_1_ treatments, respectively. These represented increases of 10.61, 6.70, and 6.27%, respectively, compared with the CK treatment. Conversely, the leaf area was the smallest under the N_1_P_2_K_2_ treatment (63.53 cm). However, the leaf area under the N_2_P_2_K_3_ and N_2_P_2_K_0_ treatments did not significantly differ, with the former showing an 18.67% increase, indicating that potassium fertilizer application at the same nitrogen and phosphorus levels was beneficial for increasing tomato leaf area.

Dry matter accumulation in tomato plants was the highest under the N_2_P_1_K_1_ treatment (310.29 g/plant), followed by 298.89 g/plant under the N_2_P_2_K_1_ treatment. Both values significantly exceeded those observed under other treatments, with increases of 59.56 and 53.68%, respectively, compared with that under the CK treatment, which had the lowest accumulation at 194.47 g/plant. Additionally, dry matter accumulation in tomatoes exhibited a trend of initially increasing and then decreasing with an increase in the amounts of nitrogen, phosphorus, and potassium fertilizers.

### 3.3 Effects of different nitrogen, phosphorus, and potassium ratios on the tomato yield

As depicted in Table 5, the average weight of a single tomato fruit was the highest under the N_2_P_1_K_1_ treatment (167.42 g), followed by 161.23 g under the N_2_P_2_K_1_ treatment. It was significantly higher than that under the CK, N_2_P_2_K_0_, and N_1_P_2_K_1_ treatments, representing increases by 26.15 and 21.49%, respectively, compared with that under the CK treatment.

**Table 5:** Effects of different nitrogen, phosphorus, and potassium ratios on the tomato yield

Treatment	Weight of a single fruit (g)	Yield per plant (kg/plant)	Plot yield (kg)	Total yield (kg hm^−2^)
N_0_P_0_K_0_	132.71 ± 4.39e	2.03 ± 0.03c	30.48 ± 0.42c	76204.00 ± 1050.74c
N_0_P_2_K_2_	150.27 ± 3.15bcd	2.45 ± 0.03abc	36.79 ± 0.52abc	91980.75 ± 1294.39abc
N_1_P_2_K_2_	149.01 ± 2.32bcd	2.63 ± 0.16ab	39.40 ± 2.43ab	98491.56 ± 6084.07ab
N_2_P_0_K_2_	147.83 ± 4.98bcd	2.37 ± 0.14abc	35.52 ± 2.04abc	88788.25 ± 5105.23abc
N_2_P_1_K_2_	153.28 ± 4.15bc	2.65 ± 0.22ab	39.70 ± 3.37ab	99241.25 ± 8432.10ab
N_2_P_2_K_2_	154.28 ± 6.50abc	2.78 ± 0.12a	41.66 ± 1.76a	104139.00 ± 4387.71a
N_2_P_3_K_2_	147.79 ± 3.30bcd	2.31 ± 0.02abc	34.70 ± 0.33abc	86.753.25 ± 818.68abc
N_2_P_2_K_0_	136.92 ± 4.10de	2.10 ± 0.11bc	31.53 ± 1.59bc	78.817.25 ± 3962.77bc
N_2_P_2_K_1_	161.23 ± 4.08ab	2.74 ± 0.16a	41.17 ± 2.37a	10.2934.88 ± 5916.02a
N_2_P_2_K_3_	146.67 ± 5.57bcd	2.53 ± 0.05abc	37.99 ± 0.76abc	94.978.75 ± 1907.91abc
N_3_P_2_K_2_	149.54 ± 6.55bcd	2.29 ± 0.05abc	34.33 ± 0.82abc	85.825.88 ± 2052.83abc
N_1_P_1_K_2_	156.65 ± 3.76abc	2.55 ± 0.14abc	38.23 ± 2.16abc	95.581.00 ± 5401.13abc
N_1_P_2_K_1_	142.12 ± 2.74cde	2.18 ± 0.15bc	32.72 ± 2.25bc	81.795.38 ± 5635.16bc
N_2_P_1_K_1_	167.42 ± 1.60a	2.85 ± 0.43a	42.72 ± 6.46a	106.803.63 ± 16,147.20a

Note: The data in the table represent mean ± standard deviation. Different letters following the numbers in the same column indicate significant differences at the 0.05 level.

The total yield of tomatoes under different fertilization treatments indicated that the yield was higher under fertilizer treatments than under non-fertilizer treatments. Among these, the highest yield was observed under the N_2_P_1_K_1_ treatment (106803.63 kg hm^−2^), followed by 104139.00 and 102934.88 kg hm^−2^ under the N_2_P_2_K_2_ and N_2_P_2_K_1_ treatments, respectively. These represented increases of 40.15, 36.66, and 35.08% compared with the non-fertilizer treatment. The interaction effect of nitrogen, phosphorus, and potassium was the strongest, followed by the interaction effects of phosphorus and potassium, and those of nitrogen and potassium, with yield increases of 20.70 and 16.51%, respectively (Table 6). The interaction effect of nitrogen and phosphorus was the weakest, with a yield increase of only 3.43%. The relative yield in the nitrogen-deficient areas was 88.32%, with a nitrogen dependence of 11.68%. The relative yield in phosphorus-deficient areas was 85.26%, with a phosphorus dependence of 14.74%. The relative yield in potassium-deficient areas was 75.68%, with a potassium dependence of 24.32%. Therefore, the order of dependence was as follows: nitrogen dependence < phosphorus dependence < potassium dependence, indicating that potassium fertilizer had a significant impact in terms of increasing yield compared with phosphorus fertilizer, whereas the effect of nitrogen fertilizer was smaller. Additionally, applying nitrogen, phosphorus, and potassium together was more conducive to increasing tomato yield.

**Table 6:** Effects of nitrogen, phosphorus, and potassium interaction on the tomato yield

Code	Treatment	Yield (kg hm^−2^)	Increased production (%)	Relative yield (%)
CK	N_0_P_0_K_0_	76.204		73.18%
PK	N_0_P_2_K_2_	91.980.75	20.70%	88.32%
NK	N_2_P_0_K_2_	88.788.25	16.51%	85.26%
NP	N_2_P_2_K_0_	78.817.25	3.43%	75.68%
NPK	N_2_P_2_K_2_	104,139	36.66%	

### 3.4 Effects of different nitrogen, phosphorus, and potassium ratios on the quality of tomato fruits

As depicted in Table 7, different ratios of nitrogen, phosphorus, and potassium fertilizer treatments significantly impacted the quality of tomato fruits. Among these, the N_2_P_1_K_1_ treatment exhibited the highest soluble protein content (3.86 mg g^−1^), followed by 3.54 mg g^−1^ under the N_2_P_2_K_1_ treatment. The soluble protein content under the N_2_P_1_K_1_ and N_2_P_2_K_1_ treatments was significantly higher than that under the no-fertilizer treatment, increasing by 69.30 and 55.26%, respectively.

**Table 7:** Effects of different nitrogen, phosphorus, and potassium ratios on tomato fruit quality

Treatment	Soluble protein (mg g^−1^)	Soluble sugar (%)	Soluble solid (%)	Vitamin C (mg 100 g^−1^)	Organic acid (%)	Nitrate (mg kg^−1^)	Lycopene (mg kg^−1^)
N_0_P_0_K_0_	2.28 ± 0.10cd	3.05 ± 0.09c	4.17 ± 0.19cde	25.82 ± 1.31f	0.23 ± 0.05b	124.21 ± 0.89bcd	12.35 ± 0.42def
N_0_P_2_K_2_	2.32 ± 0.09cd	2.66 ± 0.04de	4.20 ± 0.06cde	31.03 ± 1.04ab	0.37 ± 0.12ab	110.09 ± 3.89d	10.98 ± 0.81f
N_1_P_2_K_2_	2.09 ± 0.32d	2.53 ± 0.15e	4.30 ± 0.10abcde	29.25 ± 0.49bcd	0.47 ± 0.12a	165.88 ± 33.20a	14.90 ± 1.18cde
N_2_P_0_K_2_	3.17 ± 0.04abcd	2.99 ± 0.22c	4.23 ± 0.09bcde	32.26 ± 0.63a	0.28 ± 0.08ab	119.96 ± 0.05bcd	11.44 ± 0.09ef
N_2_P_1_K_2_	2.41 ± 0.16cd	1.79 ± 0.06g	3.73 ± 0.17f	28.97 ± 0.41bcde	0.37 ± 0.05ab	171.78 ± 5.79a	12.87 ± 0.06def
N_2_P_2_K_2_	2.87 ± 0.58abcd	3.74 ± 0.05b	4.50 ± 0.06abc	31.30 ± 0.14ab	0.33 ± 0.02ab	118.64 ± 5.74bcd	15.56 ± 0.02bcd
N_2_P_3_K_2_	2.56 ± 0.49bcd	2.15 ± 0.03f	4.13 ± 0.03de	30.07 ± 0.96abc	0.26 ± 0.08ab	157.34 ± 1.68a	17.08 ± 1.11bc
N_2_P_2_K_0_	3.25 ± 0.32abc	3.62 ± 0.07b	4.43 ± 0.07abcd	28.15 ± 0.24cdef	0.33 ± 0.05ab	125.20 ± 1.14bcd	13.83 ± 1.06cdef
N_2_P_2_K_1_	3.54 ± 0.43ab	4.36 ± 0.11a	4.60 ± 0.15a	29.52 ± 0.58bcd	0.28 ± 0.08ab	112.41 ± 1.42cd	22.44 ± 2.04a
N_2_P_2_K_3_	2.38 ± 0.01cd	2.57 ± 0.19de	3.97 ± 0.09ef	30.07 ± 0.76abc	0.28 ± 0.00ab	162.63 ± 3.94a	15.16 ± 1.87cde
N_3_P_2_K_2_	2.54 ± 0.39bcd	2.45 ± 0.07ef	4.20 ± 0.06cde	27.19 ± 0.99def	0.37 ± 0.05ab	148.65 ± 0.86ab	14.33 ± 1.93cdef
N_1_P_1_K_2_	2.84 ± 0.22abcd	2.60 ± 0.03de	4.57 ± 0.07ab	27.60 ± 0.36cdef	0.33 ± 0.05ab	142.24 ± 1.60abc	18.98 ± 1.53b
N_1_P_2_K_1_	2.95 ± 0.54abcd	2.90 ± 0.03cd	4.20 ± 0.15cde	26.50 ± 1.19ef	0.37 ± 0.05ab	146.28 ± 1.02ab	12.32 ± 0.15def
N_2_P_1_K_1_	3.86 ± 0.41a	3.16 ± 0.12c	4.40 ± 0.06abcd	26.23 ± 0.99f	0.28 ± 0.00ab	166.87 ± 1.41a	16.68 ± 0.08bc

Note: The data in the table represent mean ± standard deviation. Different letters following the numbers in the same column indicate significant differences at the 0.05 level.

Significant differences were found in the soluble sugar content of tomato fruits under different treatments. At the same nitrogen and phosphorus levels, the soluble sugar content increased first and then decreased with increasing potassium fertilizer application. Among these, the N_2_P_2_K_1_ treatment exhibited the highest soluble sugar content (4.36%), followed by the N_2_P_2_K_2_ and N_2_P_2_K_0_ treatments, which were significantly higher than that under the CK treatment, with increases of 42.95, 22.62, and 18.69%, respectively. Conversely, the soluble sugar content was the lowest under the N_2_P_1_K_2_ treatment (1.79%).

The N_2_P_2_K_1_ treatment yielded the highest soluble solid content in tomato fruits, exhibiting significant differences compared with the N_1_P_1_K_2_ and CK treatments, with increases of 10.31 and 9.59%, respectively. Conversely, the lowest soluble solid content was recorded under the N_2_P_1_K_2_ treatment (3.73%), representing a decrease of 10.55% compared with that under the CK treatment. In addition, no significant differences were observed in the contents under the CK and other treatments.

The highest vitamin C content in tomato fruits was recorded under the N_2_P_0_K_2_ treatment (32.26 mg 100 g^−1^), compared with that under the CK treatment, with a 24.90% increase. Vitamin C content under the N_2_P_2_K_0_, N_2_P_2_K_1_, N_2_P_2_K_2_, and N_2_P_2_K_3_ treatments was 28.15, 29.52, 31.30, and 30.07 mg 100 g^−1^, respectively. The vitamin C content in tomatoes increased first and then decreased with the increase in potassium fertilizer application at the same nitrogen and phosphorus fertilizer levels. This suggested that, although increasing the potassium fertilizer amount increased the vitamin C content in fruits, excessive application should be avoided.

The organic acid content of tomatoes treated with N_1_P_2_K_2_ was the highest (0.47%), whereas the content of tomatoes treated with CK was the lowest (0.23%). A significant difference in content was observed between these two treatments. In addition, no significant difference was observed in the content between the N_1_P_2_K_2_ treatment and other treatments.

The nitrate content in tomato fruits was the highest under the N_2_P_1_K_2_ treatment (171.78 mg kg^−1^) compared with that under the CK treatment, with an increase of 38.30%. Conversely, the content was the lowest under the N_0_P_2_K_2_ treatment (110.09 mg kg^−1^), marking a decrease of 11.37% compared with that under the CK treatment. Under N_1_P_2_K_2_, N_2_P_2_K_2_, and N_3_P_2_K_2_ treatments, the nitrate content was 165.88, 118.64, and 148.65 mg kg^−1^, respectively, demonstrating increases of 50.68, 7.77, and 35.03%, respectively, compared with that under the N_0_P_2_K_2_ treatment, implying that additional nitrogen fertilizer application increased the nitrate content.

The combined application of nitrogen, phosphorus, and potassium fertilizers increased the lycopene content in fruits. The highest content was observed under the N_2_P_2_K_1_ treatment (22.44 mg kg^−1^), significantly surpassing that under other treatments. This represented an 81.70% increase compared with that under the non-fertilizer treatment. Moreover, at the same nitrogen and potassium fertilizer levels, the lycopene content increased with the increase in phosphorus fertilizer amount, peaking at 17.08 mg kg^−1^ under the N_2_P_3_K_2_ treatment, marking a 38.30% increase compared with that under the non-fertilizer treatment.

### 3.5 Fertilizer model and comprehensive evaluation analysis

#### 3.5.1 Comprehensive analysis of the synergistic effects of nitrogen, phosphorus, and potassium fertilizers

Based on the “3414” field fertilizer experimental design, regression analysis was conducted on the interaction effects between nitrogen, phosphorus, and potassium using the fertilization and yield data from the 14 treatment groups. Then, a ternary fertilizer effect function equation was fitted.

\[\begin{array}{c}Y=\mbox{--}0.543\hspace{.25em}N²\hspace{.25em}\mbox{--}\hspace{.25em}2.843\hspace{.25em}P²\hspace{.25em}\mbox{--}\hspace{.25em}0.094\hspace{.25em}K²\\ \hspace{1em}+385.613\hspace{.25em}N\hspace{.25em}\mbox{--}\hspace{.25em}270.732\hspace{.25em}P+40.725\hspace{.25em}K\\ \hspace{1em}\mbox{--}\hspace{.25em}0.214\hspace{.25em}NP\hspace{.25em}\mbox{--}\hspace{.25em}0.460\hspace{.25em}NK+1.526\hspace{.25em}PK+75411.899.\end{array}]\]

According to the ternary fertilizer effect equation, the maximum application amount of nitrogen, phosphorus, and potassium was 140.49, 73.33, and 472.26 kg hm^−2^, respectively. The calculated theoretical maximum yield was 102118.84 kg hm^−2^. When the levels of nitrogen, phosphorus, and potassium were all 0, the constant term (theoretical blank area yield) was 75411.899 kg hm^−2^, which closely matched the actual yield of 76204.00 kg hm^−2^. This indicated that the fitting of the fertilizer effect function equation was relatively consistent with production reality, providing an objective reflection of the impact of combined fertilizer application on yield and fertilizer effects.

#### 3.5.2 Comprehensive evaluation and analysis of tomato quality and yield

A relatively optimal fertilizer ratio was selected using principal component analysis for comprehensive analysis. The soluble sugar content, soluble protein content, soluble solid content, organic acid content, sugar acid ratio, vitamin C content, nitrate content, tomato lycopene content, and total yield of 14 tomato treatment groups were comprehensively evaluated to select a fertilizer ratio that would produce high-quality and high-yield tomatoes. First, the original data were standardized, and then the standardized values were analyzed to obtain the eigenvalues and contribution rates of the correlation matrix (Table 8). According to the principle that eigenvalues greater than 1 can be extracted, four principal components were identified with eigenvalues of 3.54, 1.85, 1.33, and 1.15 and variance contribution rates of 39.33, 20.53, 14.76, and 12.81%, respectively.

**Table 8:** Eigenvalues and variance contribution rates of principal component analysis

Component	Total	Initial eigenvalues		Total	Sum of squared loads	
		Percent variance	Accumulation (%)		Percent variance	Accumulation (%)
1	3.54	39.33	39.33	3.54	39.33	39.33
2	1.85	20.53	59.85	1.85	20.53	59.85
3	1.33	14.76	74.61	1.33	14.76	74.61
4	1.15	12.81	87.42	1.15	12.81	87.42
5	0.57	6.28	93.70			
6	0.23	2.54	96.24			
7	0.17	1.88	98.12			
8	0.09	0.99	99.11			
9	0.08	0.89	100.00			

*F*_1_–*F*_4_ represent the scores of the four principal components, and the score coefficient equations for the four principal components are as follows:

\[{F}_{1}=\text{}0.4661{X}_{1}+0.3922{X}_{2}+0.4235{X}_{3}-0.2976{X}_{4}+0.3661{X}_{5}+0.0409{X}_{6}-0.3353{X}_{7}+0.3189{X}_{8}+0.1058{X}_{9},]\]

\[{F}_{2}=\text{}0.0375{X}_{1}+\text{}0.1905{X}_{2}+0.217{X}_{3}+0.3788{X}_{4}-0.3964{X}_{5}-0.0427{X}_{6}+0.3376{X}_{7}+0.3994{X}_{8}+0.5825{X}_{9},]\]

\[{F}_{3}=\text{}0.1336{X}_{1}-0.2134{X}_{2}+0.0833{X}_{3}+0.3522{X}_{4}-0.105{X}_{5}+0.7244{X}_{6}-0.4399{X}_{7}-0.2091{X}_{8}+\text{}0.1805{X}_{9},]\]

\[{F}_{4}=-0.2785{X}_{1}-0.0699{X}_{2}-0.3502{X}_{3}-0.4201{X}_{4}+0.353{X}_{5}+0.4546{X}_{6}+0.2599{X}_{7}+0.2431{X}_{8}+0.4006{X}_{9}.]\]

The comprehensive score for each treatment was calculated based on the values of *F*_1_, *F*_2_, *F*_3_, and *F*_4_, as well as the weights of the contributions of each principal component.

\[F=\text{}0.4498{F}_{1}+0.2348{F}_{2}+0.1689{F}_{3}+0.1465{F}_{4}.]\]

The nutritional quality and yield of 14 tomato treatments were comprehensively evaluated based on the *F* value. As shown in Table 9, the treatment with the highest comprehensive score was N_2_P_2_K_1_, followed by N_2_P_2_K_2_.

**Table 9:** Comprehensive score table of tomato nutrition quality and yield with different NPK ratios

Treatment	*F*_1_	*F*_2_	*F*_3_	*F*_4_	*F*	Ranking
N_0_P_0_K_0_	0.32	–3.03	–1.43	–0.47	–0.88	12
N_0_P_2_K_2_	–0.69	–1.12	2.09	–0.18	–0.25	8
N_1_P_2_K_2_	–2.28	1.98	0.98	–0.64	–0.49	10
N_2_P_0_K_2_	0.90	–1.65	1.36	0.74	0.35	5
N_2_P_1_K_2_	–3.46	0.79	–0.17	1.06	–1.24	14
N_2_P_2_K_2_	1.58	0.90	1.86	-0.04	1.23	2
N_2_P_3_K_2_	–0.20	–1.05	–0.78	1.93	–0.18	7
N_2_P_2_K_0_	1.03	–0.70	–0.05	–1.81	0.03	6
N_2_P_2_K_1_	4.43	1.01	0.21	0.46	2.33	1
N_2_P_2_K_3_	–1.23	–0.12	–0.20	1.56	–0.39	9
N_3_P_2_K_2_	–1.52	0.19	–0.51	–0.98	–0.87	11
N_1_P_1_K_2_	0.74	1.18	–0.64	–0.26	0.47	4
N_1_P_2_K_1_	–1.09	–0.19	–0.74	–1.74	–0.92	13
N_2_P_1_K_1_	1.47	1.81	–2.00	0.38	0.80	3

## 4 Discussion

Soil serves as the essential substrate for crop survival, with nitrogen, phosphorus, and potassium elements providing the nutrients necessary for plant growth and development. The physical and chemical properties of soil serve as crucial indicators for evaluating soil quality, directly reflecting its vitality and the status of nutrient supply. Excessive fertilization can decrease soil pH and increase EC values under experimental conditions, leading to soil acidification and increased soil salinity. This situation is not conducive to plant growth and development. The contents of soil alkali-hydrolyzable nitrogen, available phosphorus, and available potassium increase with the application of increased amounts of nitrogen, phosphorus, and potassium fertilizers. This may be attributed to the incomplete absorption of fertilizers by plants. This not only hampers the tomato fruit yield and quality but also contributes to soil salinization and fertilizer wastage, consistent with the findings of previous studies [19].

Nitrogen, phosphorus, and potassium are essential for plant growth. Plant height and stem thickness serve as crucial indicators of tomato nutrition and growth. In this study, plants treated with N_0_P_2_K_2_ exhibited the largest plant height, whereas those treated with N_2_P_0_K_2_ had the largest leaf area. This suggested that the coordination between phosphorus and potassium led to taller plants without nitrogen fertilizer, and the coordination between nitrogen and potassium resulted in larger leaf areas without phosphorus fertilizer. This could be due to sufficient nitrogen in the soil, supporting the early growth of tomato plants. Plants treated with N_2_P_1_K_1_ displayed the largest stem thickness, whereas those treated with N_2_P_3_K_2_ had the smallest stem thickness. This indicated that excessive phosphorus fertilizer might not be conducive to the increase in stem thickness of tomatoes. Within a certain range, the application of nitrogen, phosphorus, and potassium fertilizers could promote plant growth. However, excessive amounts of any nutrient led to nutrient imbalance, resulting in no growth-promoting effect or even growth inhibition [20]. Studies showed that an appropriate combination of nitrogen, phosphorus, and potassium fertilizers enhanced dry matter accumulation in potato tubers [21]. Similarly, this study revealed that plants treated with N_2_P_1_K_1_ exhibited the highest dry matter accumulation, and the interaction between nitrogen, phosphorus, and potassium significantly affected dry matter accumulation in tomatoes.

The soluble protein, soluble sugar, soluble solids, organic acids, vitamin C, and lycopene in tomato fruits are vital quality indicators that directly influence the nutritional value, taste, and commercial value of tomatoes [22,23]. The quality of tomato fruits is influenced by various ratios of nitrogen, phosphorus, and potassium, and an optimal ratio can significantly improve it [24,25,26]. This study showed that an appropriate combination of nitrogen, phosphorus, and potassium effectively improved the nutritional quality of tomato fruits. Additionally, potassium fertilizer application increased the contents of soluble sugar, soluble solids, soluble protein, and lycopene in tomato fruits to varying degrees. These contents first increased and then decreased with the increase in potassium application, which was consistent with previous research findings [27]. Additionally, excessive use of nitrogen fertilizer and prolonged application significantly elevated the nitrate content in tomato fruits, exceeding the standard limit and posing health risks to consumers.

Fertilization plays a crucial role in boosting the tomato yield. Proper fertilization practices are essential for vegetable crops to achieve high yields and maintain quality standards [28]. In the experimental setup, the N_2_P_1_K_1_ treatment led to the highest yield, with the amount of phosphorus and potassium fertilizers being below the optimal level. The impact of nitrogen, phosphorus, and potassium fertilizers on the tomato yield follows the following order: nitrogen < phosphorus < potassium, suggesting that increasing potassium fertilizer application could effectively increase the tomato yield.

Some studies showed that the pumpkin yield increased first and then decreased with the increase in the amount of nitrogen, phosphorus, and potassium fertilizers, with recommended dosages of 390.5 kg for nitrogen fertilizer, 213.8 kg for phosphorus fertilizer, and 371.3 kg for potassium fertilizer per hectare for “Jianbao” pumpkin [29]. Similarly, studies on *Angelica sinensis* suggested the optimal fertilizer application amounts of nitrogen, phosphorus, and potassium fertilizers to be 81.15, 80.67, and 29.78 kg hm^−2^, respectively, resulting in a maximum seedling transplanting yield of 1932.52 kg hm^−2^ [15]. The ternary quadratic fertilizer function equation fitted in this “3414” fertilizer experiment expressed the relationship between the total tomato yield and the application of nitrogen, phosphorus, and potassium fertilizers. Based on this equation, the calculated application rate was 140.49 kg hm^−2^ for nitrogen fertilizer, 73.33 kg hm^−2^ for phosphorus fertilizer, and 472.26 kg hm^−2^ for potassium fertilizer, with a fertilizer ratio of 1:0.50:3.05. The highest expected yield was projected to be 102118.84 kg hm^−2^.

## 5 Conclusions

Fertilization significantly impacts both the quality and yield of tomatoes. Applying nitrogen, phosphorus, and potassium fertilizers in various proportions can enhance soil fertility, stimulate tomato plant growth, and augment both the quality and yield of tomatoes. A comprehensive analysis revealed that the most suitable fertilization treatment under the experimental environment and planting conditions was N_2_P_2_K_1_. Therefore, N_2_P_2_K_1_ was recommended as the optimal fertilization formula for producing high-quality and high-yield tomatoes in the experimental area, with the amount of nitrogen fertilizer being 197.28 kg hm^−2^, phosphorus fertilizer being 88.75 kg hm^−2^, and potassium fertilizer being 229.80 kg hm^−2^. This approach was conducive to achieving efficient and high-quality tomato production. However, the yield and quality of tomatoes were affected by various factors besides fertilization, necessitating further studies on other aspects.
